# Emotional violence by teachers toward students: the adultcentric bias hypothesis

**DOI:** 10.3389/fpsyg.2026.1849830

**Published:** 2026-06-16

**Authors:** Daniele B. Santana

**Affiliations:** Nova School of Science and Technology, Faculty of Science and Technology, New University of Lisboa, Caparica, Portugal

**Keywords:** Adultcentrism, bias, bleak pedagogy, emotional violence, teachers

## Abstract

Emotional violence (EV) from teachers toward students is a serious and underexplored issue in school settings. This study aims to review current research on EV, explore the concepts of Bleak Pedagogy (BP) and Adultcentrism as conceptual frameworks, and explain the underlying causes of emotionally violent behaviors. Given the global prevalence of EV, we infer that it may be influenced by embedded structural biases in adult–child relationships, rather than from individual traits. Furthermore, it is proposed that these systemic biases and attitudes may act as key predictors of EV from teachers toward children. Finally, empirical investigations into teachers’ pragmatic and paradigmatic beliefs in relation to students’ self-reports of victimization are suggested to test this hypothesis. Further implications of this perspective and directions for future research are discussed.

## 1 Introduction

This article addresses a concerning subject—the emotional violence (EV) that teachers perpetrate against their child-students. This form of violence can be referred to using other terminologies, such as psychological violence, emotional abuse, and verbal abuse. Emotional violence (EV) is a concept that applies not only to various relationships but is also noted in a only systematic review when it occurs in teacher-student interactions (Scharpf et al., 2023). It was, therefore, selected for use in this study. In general, research points that when EV occurs within the family context, it is more closely associated with psychiatric sequelae than physical abuse and serves as a more significant precursor to dissociation than sexual abuse (Teicher et al., 2006).

The socioecological perspective (Bronfenbrenner, 1979) presents a multi-level system in which human development is a dynamic interaction between personal and situational factors. It is organized in four nested systems: micro, meso, exo, and macrosystem. The microsystem refers to individual-level interactions; the mesosystem refers to interactions between microsystems; the exosystem encompasses indirect environments, such as institutional actions; and the macrosystem refers to broader societal values and patterns. From this perspective, teacher–student interactions are highlighted as an important microsystem in which children participate.

Children who attend schools tend to spend a large amount of time there, making teacher–student interactions an important microsystem in children’s lives. This relationship affects their emotional and social development, school liking, and adjustment in school transitions (Longobardi et al., 2019; Thornberg et al., 2023). Teachers stand as figures from whom students cannot withdraw, and their use of EV is prejudicial and can reinforce violent behaviors (Scharpf et al., 2023; Longobardi et al., 2015).

Although it has not been researched as extensively as other forms of violence against children or other forms of school violence, EV from teachers toward students is a growing concern among scholars and policymakers. Over the past 20 years, there has been an increase in research on this topic, as well as its recognition in official documents, such as those from the United Nations Educational, Scientific and Cultural Organization (UNESCO, 2024). The known impacts of this type of violence on children’s well-being and development are outstanding.

Children who experience emotionally victimization by teachers often show more behavioral difficulties and engage in delinquent behavior (Aluede et al., 2012; Kızıltepe et al., 2020; Nearchou, 2018). Research has also shown an association between teacher-perpetrated EV and various outcomes, including somatic complaints (Hyman et al., 1988), depressive symptoms (Pottinger and Stair, 2009), anxiety (Krugman and Krugman, 1984; McEachern et al., 2008; Aluede et al., 2012), and negative school perception and performance (Datta et al., 2017; Fromuth et al., 2015; Kızıltepe et al., 2020; Forsberg et al., 2023). Therefore, EV perpetrated by teachers should be of major concern.

The approach adopted critically examines the current literature to present a novel, broader perspective on its predecessors. Current research has failed to explain the root causes of certain behaviors because it generally focuses on individual characteristics rather than on structural systems of oppression. Research has aimed to understand the victimization of students by examining possible precursors in their personal traits and behaviors (e.g., Lee, 2015). Inadvertently, this can lead to the re-victimization of children, suggesting that there is something inherent in them that makes them susceptible to victimization by their teachers (Gusfre et al., 2022; Scharpf et al., 2023).

Regarding the teacher profile, only a limited number of studies have been conducted, and there is no consensus among them. Mainly covering individual traits, such as gender, personal history, and years of experience (e.g., Khoury-Kassabri, 2012; Kızıltepe et al., 2020; Twemlow et al., 2006), this body of research has limits in establishing a conclusive framework for how EV persists across cultures, teacher profiles, and educational contexts. There is an outstanding gap in understanding the factors that underlie teachers’ actions. Current findings have not adequately explored how teachers’ biases and worldviews, shaped by broader societal constructs, may lead them to act in emotionally violent ways.

The study assumes an inductive approach and proceeds in four interconnected steps. First, the current literature on emotional violence perpetrated by teachers toward students is critically examined. Second, the concept of Bleak Pedagogy (BP) is explored as a framework closely related to EV. Third, Adultcentrism is introduced and discussed as BP’s conceptual counterpart and a belief system that may sustain violent adult practices. It discusses how this interpretation offers a novel perspective on the underlying causes of EV in school settings, reframing its causes as rooted in pedagogical and societal worldviews. Finally, the possibilities for hypothesis testing are presented, followed by broader implications for future research, teacher training, and educational practices. By centering structural rather than individual explanations, it is focused not merely to document another form of school violence, but to expose the deeper cultural logics that make emotional violence seem normal and even necessary, aiming to challenge assumptions and invite a rethinking of how emotional harm in education is understood, addressed, and ultimately prevented.

## 2 Current evidence

This study develops a theoretical argument that provides a new lens for understanding emotionally violent behaviors from teachers toward students and a potential explanation at the structural level. Evidence from the literature, predominantly examining individual-level characteristics of students and teachers, is identified and its results critically discussed.

The critical review approach was selected since it enables evaluation of prior literature and provides a foundation for developing conceptual innovation and future testing (Grant and Booth, 2009). Given the study’s theoretical purpose and aim of a broad, critical examination of findings, citation chaining within a purposive snowballing approach (Wohlin, 2014) was employed to expand the literature. Initial searches were conducted in May 2025 across multiple databases for English results, such as Scopus, American Psychological Association (APA), and Web of Science, using various keyword combinations due to the broad spectrum of terms: emotional abuse, emotional violence, psychological abuse, psychological violence, verbal abuse, maltreatment, teacher–student, and teacher bullying. A complementary search was conducted in Capes and RCAAP for studies in Portuguese. The results were limited to peer-reviewed publications in English or Portuguese. Titles were screened to identify those meeting the eligibility criteria that required studies to address emotional violence perpetrated by teachers or school staff toward children within the school setting. Studies on peer-violence, from students toward teachers, outside of school, or only referring to other forms of violence, were excluded. After removing duplicates, an initial set of identified literature reviews (Scharpf et al., 2023; Gusfre et al., 2022) and 10 articles was selected to cover different authors and countries. Backward and forward citation chaining were applied to three interactions using the same eligibility criteria but with no date restrictions. After full-text reading, 35 articles were included. The following sections organize and critically review the main contributions from these studies.

### 2.1 The hidden violence

Among the physical, sexual, and psychological violence children are exposed to, either at home, in the community, or at school, EV presents a difficulty in identification, as psychological maltreatment faces several inconsistencies regarding conceptual, legal, and operational definitions (Baker, 2009). A conceptualization broadly accepted for defining EV is as disciplinary or motivational practices, verbal or non-verbal, that do not rely on physical violence, and cause psychological damage to children (Hyman et al., 1988; Hyman and Snook, 1999; Scharpf et al., 2023).

Emotional violence from teachers can range from overt actions to more subtle behaviors. While some are directly identifiable, others can be less obvious and “leave space” for argumentation. Research documents overt EV actions, such as teachers shouting, screaming, or yelling at their students (Shumba, 2002; Gazioğlu, 2021), as one of the most common forms of EV in some studies (Fromuth et al., 2015; Kızıltepe et al., 2020). In addition, externally visible for observers, it can manifest itself as cursing (Benbenishty et al., 2002; Lee, 2015) and swearing at students (Nearchou, 2018; Gazioğlu, 2021), and humiliating them by calling derogatory names such as “stupid” or “idiot” (Shumba, 2002; Lee, 2015). Other reported forms involve the use of sarcasm, humiliation, and intimidation (Benbenishty et al., 2002; Shumba, 2002; Gazioğlu, 2021), which is commonly witnessed by other students.

The literature also identifies EV as isolating, ignoring, and/or rejecting a student (Aluede et al., 2012; Fromuth et al., 2015; Longobardi et al., 2015; Nearchou, 2018), dominating behaviors (Longobardi et al., 2015), terrorizing (Aluede et al., 2012), and threatening (Gazioğlu, 2021; Kızıltepe et al., 2020). As the UNESCO (2024) defined, “Related to education, this can be manifested by such actions as humiliation, intimidation, and threats, or isolating, rejecting, ignoring, excluding from a group, spreading rumors, or name-calling.” This range of documented experiences and definitions reflects EV’s multifaceted nature, which can manifest in overt actions or in subtler, relational forms. The lack of physical evidence and the manipulative, implicit nature of the evidence play a significant role in its identification and reporting. Since they are harder to identify, there are also fewer reports and charges against their perpetrators.

Some authors have chosen the term “teacher bullying” when analyzing violent actions from teachers, which can be physical, relational, or psychological. The traditional definition of bullying by Olweus is that a “student is being bullied or victimized when he or she is exposed, repeatedly and over time, to negative actions (…) [while] there should also be an imbalance in strength (an asymmetric power relationship)” (Olweus, 1997, p. 496). Relevant works have used “teacher bullying” and are included in this study. While presenting meaningful insights, including on the power imbalance in teacher–student relationship, the “teacher bullying” framework cannot address situations in which the victims may vary. Given that EV encompasses a behavioral model that can be repetitive and constant, occasional yet particularly incisive, or situational (Longobardi et al., 2015), the ‘bullying’ framework risks compartmentalizing rather than integrating the diverse manifestations of EV.

Emotional violence is frequently normalized in schools and accepted as a way to discipline children (Shumba, 2002). Teachers tend to justify their behavior as a response to provocation and unacceptable student behaviors, citing “motivation” or as part of instruction/teaching (McEvoy, 2005), with some stating that EV can result in a better learning environment (Lee, 2015). Across countries, child protection legislation sometimes even accepts corporal punishment as an effective means of addressing discipline and behavioral problems (Dubanoski et al., 1983). Scharpf et al. (2023) observed that in some countries in Asia and Africa, where physical punishments in schools are not legally banned, they are the predominant form of school adult violence toward students. European, American, and Middle Eastern countries that have banned these practices report a relatively higher rate of EV. These differences indicate that legislative changes can, to some extent, protect children. In contrast, the values that guide disciplining children violently are present and manifest in forms that are legal or not perceived as violent.

Teachers often do not perceive their own or their colleagues’ emotionally violent behaviors. A study indicated a lack of recognition of the hostile, conflictual relationship between teachers and their students (Longobardi et al., 2017). Even in schools committed to anti-bullying programs, teachers demonstrated low awareness of teacher bullying^1^ and greater tolerance toward socioemotional bullying perpetrated by their colleagues against students (Zerillo and Osterman, 2011). At the institutional level, earlier studies have shown that principals condone teachers’ abusive behavior, interpret students’ compliance and quietness as evidence of teaching efficacy (Krugman and Krugman, 1984). Emotional violence from teachers has been documented as underreported (Shumba, 2002) and, according to the UNESCO (2024), one in four teachers do not recognize teachers’ EV as violence. These findings emphasize the difficulty of recognition and the higher tolerance for teachers’ EV.

Ultimately, the inconsistency in terminology may affect teachers’ understanding and ability to combat EV. A broad range of actions falls within the EV spectrum, and not all are easily identifiable by a victim, teacher, or bystander. Evidence suggested that even teachers trained to prevent bullying may fail to recognize it in their own or other teachers’ practices. A critical concern is how these behaviors can still be understood as acceptable, necessary, and motivational—a justificatory discourse that employs the protection rhetoric to legitimize abuse and control.

### 2.2 Protection rhetoric as justification

The authority rhetoric as a means of protecting children has not shown to come to reality, as evidence has pointed out that the “protection of children” speech, while based on adult power, can actually lead to double victimization of children. In school bullying situations, boys who were bully victims (victims and perpetrators of bullying among peers) had the highest rates of victimization by school staff, followed by bullies. In contrast, the ones not involved in peer bullying reported lower levels of victimization by school staff (Khoury-Kassabri, 2009). Another study has shown a greater risk of being victimized by teachers for those students who were victimized by their peers, impacting their school attendance due to fear of violence (Khoury-Kassabri, 2011). It is possible to conclude that order and discipline in school settings appear to overcome the actual protection of children, failing to protect them and inflicting double victimization.

As in general bullying situations, the asymmetric relationship between the school actors can be one of the structures that make bullying happen. Freire’s theoretical claim that “when education is not liberating, the dream of the oppressed is to become the oppressor” (Freire, 1968/2014) seems to find empirical reality. Teachers who identified bullying behavior toward students in their colleagues pointed out that one of the types is a teacher who is a bully victim. The teachers who are more likely to experience bullying from students and bully their students are the ones who were victimized when young (Twemlow et al., 2006).

The quality of teacher–student relationship was shown to be related to verbal and relational bullying victimization among peers, both predicting and being predicted by it, resulting in victimization cycles between the teacher, student, and students, both as bully victims and bullying victims (Marengo et al., 2021; Forsberg et al., 2023). The adult, power-seeking protection does not realize its purpose, yet it is entangled in the experiences and deeper beliefs that educators carry.

Despite the current efforts some countries have set into place with their school policies, teacher emotional violence is a worldwide situation. While the diversity of sampling, used scales, and terminology must also be taken into account, reported rates range from 0.6% (Sweden) to 98% (Italy) of victimized children (Scharpf et al., 2023). Countries with strict anti-bullying policies that include teachers and staff as perpetrators may present lower but still concerning numbers. As in the case of 1.6% of Norwegian students who reported in 2019 having suffered teacher bullying at least two or three times a month (Gusfre et al., 2022), a number that has increased to 4.9% in grade 10 in 2023 (Norwegian Directorate for Education and Training, 2024). Other studies found higher prevalence of teacher EV, for example, 18.2% in South Korea (Lee, 2015) and 86.2% in Nigeria (Aluede et al., 2012). A Turkish study found that yelling was the most excused behavior for teachers and parents. In the same study, children stated that resorting to violence, instead of non-punitive strategies, was excused when used by their teachers to deal with classroom issues (Gazioğlu, 2021). Showing, therefore, that EV is not only present but can be excused and justified to be used by teachers and staff in the educational setting.

Protection does not seem to motivate teachers to act accordingly, and protection programs have yet to fully achieve the goal of ending teacher violence. As in general bullying situations, the asymmetric relationship between the school actors can be one of the structures that drives bullying to happen.

### 2.3 Student profile: misguiding the view

Recent reviews have found that most studies assess children’s characteristics that predict teacher victimization. From Gusfre’s work (Gusfre et al., 2022), we note that according to 14 studies, boys are at a higher risk, while three pointed at a higher prevalence among girls (Elbedour et al., 1997; Datta et al., 2017), although other studies have found no differences concerning gender (James et al., 2008; Chen et al., 2011). Regarding the form of violence, girls appear to be suffering greater violence in the forms of neglect and verbal abuse (Ali et al., 2013). Understanding from early feminist writings, gender stereotypes in girls’ socialization aim to mold them into feminine ideals of subordination and care (Beauvoir, 1949/2011). Even beyond traditional binary categories, gender categories are built upon discourse and practices (Butler, 1990). Those stereotypes are reinforced at school, as when teachers tend to perceive boys as misbehaved, physically and emotionally stronger (Skipper and Fox, 2021), boys’ higher prevalence of victimization can be due to their learned boy-behavior and the adult perception of those as more disruptive to the school setting. Different results in a few of the studies can be related to cultural aspects of where the research took place, and the type of bullying victimization girls suffered.

From a gender perspective, relationship quality with teachers shows that female teachers’ perceptions of their relationships with students vary significantly. Their relationship with girls is pointed as closer and with higher dependency, while more conflictive with boys (Quaglia et al., 2013). Whereas boys seem to face a harder time in school regarding their relationships with teachers, self-perception, and victimization, the different findings are yet to answer the questions. On the other hand, the gender prediction of victimization and relationship quality did not address side characteristics and underlying beliefs that could predispose teachers to target boys more often.

A colonialist and biased view of the world can manifest within the school setting. A large study on Israeli schools found that Arab children suffer more victimization from school staff, both physical and emotional, in almost all bullying involvement scenarios (Khoury-Kassabri, 2009); in the US, Black male students were found to be significantly more physically punished than white students, and extremely more than white girls (Gregory, 1995). Also in the United States, of the 88% of students who reported being psychologically mistreated, at least once, by an adult in school, 20% were made fun of due to their race or skin color (Dupper and Whitted, 2007). In that regard, a study involving preschool and kindergarten students and teachers has shown that having students of different ethnicities can magnify teacher–student relationship conflicts (Saft and Pianta, 2001). The teacher’s perception of children, their heritage, and culture as more similar to their own seems to play a significant role in their actions. While students’ characteristics can predict relationship quality and the likelihood of teacher victimization, it is the teachers’ values and beliefs that should be addressed.

Current research has failed to explain the root cause of such behaviors due to its general focus on children’s individual characteristics rather than structural systems of oppression. Roughly three times as many studies have sought to understand EV victimization by examining potential precursors in students’ traits and behaviors rather than in teachers’ traits. Children are, unintentionally, positioned as the cause of teachers’ violent behaviors, while the beliefs that lead them to target some groups are not overlooked.

### 2.4 Surface individual traits: teacher profile

When addressing the teacher profile, only a limited number of studies have been conducted, and there is currently no consensus among them. While it mainly covers individual traits, such as gender and years of experience, it still fails to provide a conclusive framework for how teacher EV persists across cultures, teacher profiles, and educational contexts.

The findings regarding teacher gender were not unanimous. According to Scharpf et al. (2023) review, some researchers concluded that female teachers are more likely to use emotional violence (Fromuth et al., 2015; Montuoro and Mainhard, 2017) while no gender difference was found in others (Khoury-Kassabri, 2012; Kızıltepe et al., 2020; Theoklitou et al., 2012). Teacher trainees and teachers believed female teachers to be more likely to commit emotional abuse against students (Shumba, 2002), yet Pottinger and Stair (2009) found that male teachers were more abusive. The diverse results can be explained by the research conducted in different socio-cultural contexts and by differences in measurement and data-gathering methods.

Teachers’ subjective beliefs appear to play a strong mediating role in their use of physical and emotional violence. Their self-efficacy perceptions were mediated by their beliefs regarding their use of violence and their students’ abilities, “shaping the disciplinary beliefs of teachers, which then affects teachers’ tendency to use physical and emotional violence toward their students” (Khoury-Kassabri, 2012). It is noted that teachers who engage in bullying behavior can present personal characteristics such as self-confidence and intellectual capacity, with high acceptance among teacher peers and school administration. Such qualities, along with others’ perceptions, can position them as accepted leaders in the school and contribute to the continuation of bullying behaviors (Burris and Snead, 2018). Teachers’ internal states appear to shape the interpretation of students’ misbehavior and can lead to more impulsive and aggressive responses (Montuoro and Mainhard, 2017). The perceptions of self as the peers and authority perceptions seem to play a significant role in violent behaviors toward students, yet are not capable of acting as a predictor for such.

Although understanding child development can be seen as a means to improve teachers’ efficacy in the classroom and in student management, research points in a different direction. Few studies were found to explore years of experience or educational level concluding that teachers with 5 or more years of experience are more likely to commit bullying toward students (McEvoy, 2005) and as the higher educational level, more teachers made use of physical violence (Khoury-Kassabri, 2012), but also no relation between years of experience and self-report use of EV by teachers (Khoury-Kassabri, 2012). It is important to note that social desirability factors can bias self-report data collected from teachers. Nevertheless, the present results resonate with current criticisms of developmentalism (Biswas et al., 2023), which posits a universalist model of development and progress, to which children are also framed. Knowledge of those child development theories may, instead of improving teachers’ relationships with students, misguide their actions toward an authoritarian perspective, reinforced by their perceived superiority over children.

It seems reasonable to assume that there are broader systems of beliefs, beyond the objective levels, that motivate teachers to make use of emotional violence in the classroom.

### 2.5 Ontological subordination: power imbalance

The school, as established, is the institutional instrument for controlling and disciplining children, for turning their bodies into more docile ones, within a structure in which the adult figure embodies authority, control, power, and order (Foucault, 1975/1999). For decades, children have been conceptualized as incomplete human beings, “not finished but in a state of becoming” (Arendt, 1961, p. 185), who will achieve their full being as productive adults in the capitalistic system (Shabel, 2024). A vision aligned with neoliberal capitalism that trains new workers to perform the same tasks as their parents, more efficiently (Gatto, 2010). Freire’s (1968/2014) concept of “banking education” reflects the dynamics of children as empty boxes, in need of receiving adult knowledge to become full. Their economic utility, and productivity measures their worth within the system until they are too old to be productive. Oldness is another age hierarchy that merits its own discussion.

Teachers are positioned with greater power than students in terms of hierarchy, but also to hold knowledge, higher social status, and concrete power over routines, actions, and access to “privileges” (Fromuth et al., 2015; Scharpf et al., 2023), driving teachers to consciously or unconsciously subordinate students, generating a power gap among them (Eriyanti, 2018). Pertinent to Bourdieu’s symbolic violence and the unawareness of those who act and suffer it (Bourdieu, 1996, p. 17), this power gap is considered one of the possible causes for verbal abuse. This structural design aligns with the top-down structure of formal educational systems, reinforcing the power that teachers retain.

Supported by the knowledge transmission paradigm, adults have power and rights over children, such as withdrawing privileges and ostracizing, among other violent punishments, both physical and emotional. In contrast, children’s perspectives can be ignored and their bodies controlled. Having the government, school boards, and teachers create curriculum and content to fill the “empty box” of a child with adult knowledge, without taking into account the child’s perspectives, reproduces hierarchical power.

Power relations and hierarchy are often presented as natural in the schooling system, and permeate the environments children encounter. However, development and learning are better supported when power is redistributed, thereby increasing children’s control (Bronfenbrenner, 1979, p. 58). Therefore, the perceived standard structure of educational relationships may be counterproductive to its foremost objective of supporting learning and development.

### 2.6 EV conclusion

A critical review of current research on teacher-inflicted EV toward students pointed to a lack of consensus and understanding on what is compelling teachers to act in emotionally violent ways toward their children-students.

The terminological and definition variations point to a difficulty in understanding and combating emotionally violent behaviors. The protective and motivational discourses that justify the use of EV do not withstand scrutiny/lack empirical support, as the outcomes are associated with suffering and lower academic performance. Moreover, while some children’s characteristics can make them more vulnerable to victimization, and teachers’ individual histories and profiles can indicate a tendency to use EV, these factors do not provide a comprehensive framework for combating EV among teachers.

Even considering cultural and structural differences among the countries where the research was conducted, with specific socio-cultural structures, school climates, educational systems, and other singularities, EV from teachers is present to a major or minor extent across countries, underscoring the need for further evaluation to understand it better.

The structural hierarchical characteristics of the school system seem to be a promising indicator of causes for emotional abuse from teachers, yet further investigation is necessary. Another approach appears necessary if we want to address the root causes of such behaviors to improve child protection and rights. In this respect, we recur to broader, implicit beliefs and mechanisms to understand it.

## 3 Underlying socio-cultural mechanisms

**Figure 1:**
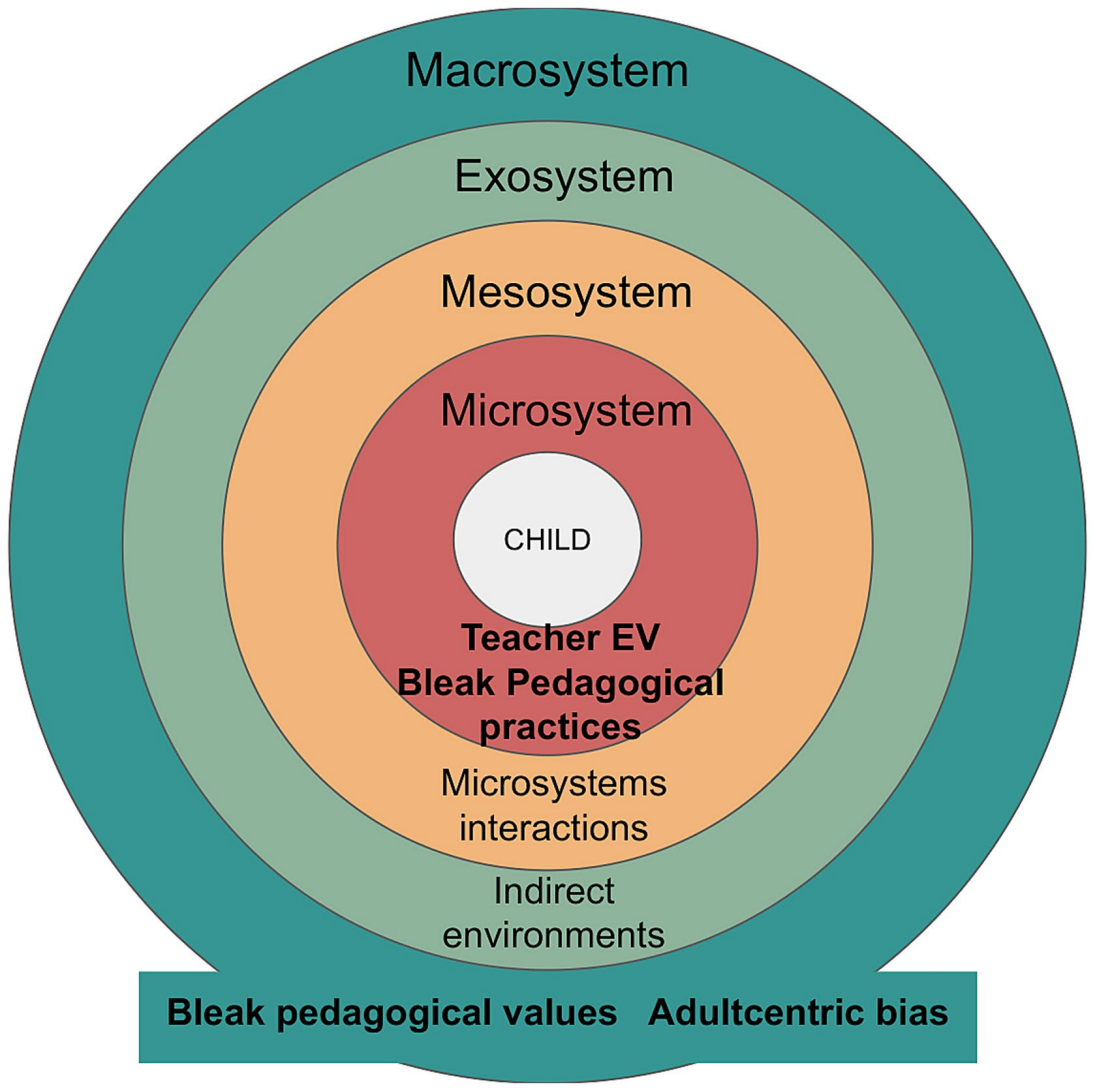
Teachers’ EV research and hypothesis situated in Bronfenbrenner’s (1979) socio-ecological systems.

The socio-cultural mechanisms are present in all our relationships, yet can go unnoticed. Supported by the socio-ecological perspective (Bronfenbrenner, 1979), we find that most studies have focused on the microsystems and mesosystems; no study has addressed the macrosystem level, leaving a gap in the empirical testing of the beliefs that structure power imbalances between teachers and students. Aiming to address this gap, the concepts of Bleak Pedagogy and Adultcentrism are positioned, as shown in Figure 1, and developed in the next sections.

### 3.1 Bleak pedagogy—new term to address old behaviors

The term Black Pedagogy, derived from the original term Schwarze Pädagogik, was introduced by Rutschky (1977) as a retrospective definition of values and disciplinary practices of the eighteenth and nineteenth centuries. Recent research from Florio et al. (2022) rescued the term firstly in its direct translation as Black Pedagogy and has drawn upon several authors (e.g., Miller, 1980, 1983; Brokate, 2005; Kühn, 2014; Rutschky, 2015) when mobilizing this concept as an aiming for bourgeois qualities, as diligence, humility, and subordination, thought the acceptance of an hierarchical concept of family, and societal institutions as the school, in a “child-rearing culture” This dynamic maintains the cycle of subtle and explicit violence across generations.

Avoiding the good–bad opposition, it should currently be referred to as Bleak Pedagogy to shift the perspective from a judgmental good–bad framing adults, teachers, and parents, toward an understanding of the frustration and desolation adults may feel when inserted into an authoritarian structure and facing challenging situations. When faced with those situations, adults are struck by the perception of a lack of options, believing they have no other way; even if their actions are unpleasant, they are understood as necessary for the child’s own good (Florio et al., 2025). As the violence cycles, the intergenerational transmission of violent responses to difficult situations is based upon learned behaviors and authoritarian structures (Twemlow et al., 2006), replicated by children, adolescents, and adults as the only possible/learned response.

Beyond the reasons presented by the authors, the alteration from “Black Pedagogy” to “Bleak Pedagogy” also refrains from the common racist societal construction of relating black with bad, immoral actions (Alter et al., 2016) and avoids falling into the racist connotation of black and blackness in language for terms denoting bad actions, behaviors, and practices (Lingayah and Kelly, 2023). The change also moves the negative concept further away from anti-racist practices as African-Centered Pedagogy, pro-Black Pedagogy, and Critical Black Pedagogy (Murrell, 2002; Pitre et al., 2022; Bicknell-Hersco, 2025).

In peer-bullying situations, the authority of teachers and their role as models of behavior appear to be among its causes. The direction of bullying victimization is not clear, yet a recent Norwegian study observed that an increase in offline peer-bullying victimization is correlated with teacher bullying victimization. Data show that this association was stronger among younger children, consistent with the understanding that children separate themselves more from adult models as they reach adolescence (Gusfre et al., 2024). While no causality could be determined, given that teachers model behavior in the classroom, it is argued that when teachers victimize students, they will act similarly, by bullying their colleagues. This explains a relatively small number of bullying cases, yet it also addresses the least-explored relationship.

The use of the term BP may refer to the discrepancy in data collection of teachers’ violent practices report and student victimization report. As noted by Burris and Snead (2018), teachers reported almost no bullying behavior toward their students; however, when university students recalled their school experiences from early schooling through university, almost all of their narratives fit the bullying definitions, even though those definitions had not been presented to them previously. Emotional violence was reported by around 98% of middle school Italian students, while only around 2% of teachers reported acting in emotionally violent ways (Longobardi et al., 2015). The Bleak Pedagogy framework can embrace systemic patterns while acknowledging that teachers can be, themselves, victims of intergenerational violence and oppression cycles, in school violence, and lack of perception on “what is emotional violence?”

Bleak Pedagogy can be understood as tools of adult power that control and diminish children through acts that silence them. An attempt to acknowledge both physical and psychological punishments, whether in-home or at school, the harm they inflict, and the embedded power dynamics is the use of the term “corporal punishment and/or discipline (CPD)” (Lokot et al., 2020). Kühn (2014) highlighted the intention to break a child’s will through demeaning actions to shape children into ideal values based on society and the adults involved in their education (as cited in Florio et al., 2020b).

As noted, addressing violence from teachers toward students is a concern, while its definitions, identification, and reporting by the school actors are still insufficient (e.g., Baker, 2009; Shumba, 2002). Adding to these findings, difficulty in recognizing subtle maltreatment in the classroom was correlated with greater agreement with BP, which in turn was associated with more rigid teaching styles and agreement with “old-fashioned” educational methods (Florio et al., 2022). The reported transgenerational transmission of abusive behaviors (Twemlow et al., 2006) can be one of the causes for such agreement with “old-fashioned” methods of education. As pointed out by Hyman et al. (1988), “in some cases it is clear that both physical and emotional abuse and maltreatment are part of a syndrome of overly severe disciplinary practices in schools.” The recognition and report of emotionally violent behaviors toward students is, therefore, entangled with a broader system of values, beliefs, and intentions that can go unnoticed even for their perpetrators.

Regardless of personal and cultural beliefs, Bleak Pedagogical practices extend beyond educational and motivational practices, as they can infringe on global and local legislation. Given adults’ control and power over children, those practices, called “old-fashioned” (Florio et al., 2022), also constitute illegal actions under child protection legislation, such as the Convention on the Rights of the Child (United Nations, 1989). They can also violate national laws, such as the Brazilian “Children and Adolescents Statute” that states the “inviolability of the physical, mental, and moral integrity of children and adolescents” and the “duty of all to ensure the dignity of children and adolescents, protecting them from any inhumane, violent, terrifying, vexatious, or embarrassing treatment” (Brasil, 1990). Therefore, regardless of being considered acceptable in some cultures and societies, such practices are broadly understood as violence against children.

Supporting that while teachers may avoid/not accept detrimental practices, they agree and carry the values that support behaviors as such. As clear as the detrimental practice is, the more it is recognized and further abandoned by adults, being the emotional violent behaviors the ones with lower decrease, when compared to physical ones. Furthermore, Florio’s results have shown a stronger agreement with BP values than practices (Florio et al., 2022). Combining the greater decrease in physical maltreatment with a lower decrease in psychological maltreatment, and their relationship to power imbalances between teachers and students, it is possible to relate these practices to the persistence of educational values and objectives (Florio et al., 2020a, 2020b). Aligned with the findings, emotional violence is recognized as under-estimated, under-reported, and under-recognized (Malo et al., 2017), showing the necessity to identify the link between actions, values, and perceptions. By analyzing such behaviors, it is possible to advance research, determine whether those values are present across cultures and societies, and uncover the roots of teacher emotional violence.

One concept related to Bleak Pedagogy and that appears as a possibility for predicting behaviors is Adultcentrism, a bias and worldview. Current results with the BP Scale have shown that the agreement with values and beliefs on BP and “education of children over time” was higher than with its methods. Furthermore, the adultcentric view of children and the adult–child relationship were shown to be significant predictors of BP practices (Florio et al., 2022). Considering BP as an “umbrella term” that can translate beliefs and practices parallel to EV, bullying, and/or verbal abuse, the adultcentric bias could be, therefore, the underlying cause of teachers’ EV that are yet to be fully understood in their predictors and motivations.

### 3.2 Adultcentric bias explanation

Historically, it is in the late twentieth century that Goode claimed to have coined the term adultcentric regarding the commonsense beliefs about children from a societal framework where:

Adult-authored models of socialization and education which are found massively in our studies of children and which present children as: (1) essentially requiring the intervention of adult society to acquire basic human competencies; (2) tabula rasa—bundles of potentialities to be nurtured until their full and competent expression in adulthood; and, (3) as growing in relatively clear developmental stages (physical, sensorimotor, moral, linguistic, cognitive and social) toward adulthood (Goode, 1986, p. 84).

It must be noted that other meaningful and more recent contributions to this concept have pointed out how the fundamentals of our understanding of child development can produce and reproduce an adultcentric bias (Matusov and Hayes, 2000), carrying the belief in children’s incompletitudeness and incompetence (Petr, 1992). Our own adulthood blurs the lens on viewing children by defining them in opposition to adults, gathering the conceptualization from Mackay (1974), Grotberg (1977), and Cannella (1997), to point out that children are “small in size, emotionally unstable, irrational, illiterate, egocentric, dependent, and amoral; adults instead are: large in size, emotionally stable, rational, literate, sociocentric, independent, and moral (adapted from Grotberg, 1977, p. 392),” describing children as “incomplete, immature, irrational and lacking in skill or knowledge, whereas adults are seen as complete, mature, competent and in control” (as cited in Florio et al., 2020a).

The image of the child is, therefore, constructed as an absence, a lack, and the opposite of the fully accomplished adult. This imagistic construction legitimates and naturalizes hierarchy and top-down relationships and structures within society, families, and schools. Children are systematically subordinate to adult supremacy, diminishing their capabilities and limiting their social power (DeJong and Love, 2015). Those structures, present in the definitions of bullying, can also be the ones that allow and predict forms of emotional violence that, while not repetitive on the same target as the bullying ones, are present in the teacher–student relationship.

Positioning adults as the center and standard of humanity is not novel and can be seen as part of a broader paradigm of hierarchical thinking. Goode has identified Adultcentrism as a form of ethnocentrism, as both operate through “assumptions that privilege one group’s perspectives and standards, while presenting similar negative consequences” (Goode, 1986). Supporting itself on this view, children face similar consequences to subjugated peoples, having their perspectives and culture unaccepted, their actions misjudged, their strengths undermined, and their self-determination limited by adults (Petr, 1992). Akin to what was done to colonized peoples, holding a perspective of adults as the standard for humankind, children are positioned as incomplete and inferior humans that must accept the adult position of missionaries to indoctrinate and help them to fulfill their humanity through teaching them how to function in society, disregarding their own culture and beliefs (Goode, 1986). Educational institutions enact society’s paradigms as mechanisms for colonizing young people, exercising the microphysics of power over children to docilize their bodies (Foucault, 1977/1984; Liebel and Meade, 2024).

Other authors indicate how the parallels between Adultcentrism and ethnocentrism become more evident when historically analyzing anti-Black racism and European colonial violence, shifting our understanding of their origins. This positioning of certain groups as “child-like” has justified their subjugation by European countries. Using the argument of their existence in the “childhood of humanity,” they are identified as children in need of discipline, guidance, correction, and insertion in the “developed” culture, through naturalization of violence and servitude, as it is accepted to practice with children (Rollo, 2018). This perspective shows that it was the bias in how we see children and how they can be “tamed” that allowed the same logic to apply to people, pointing to the cross-situational importance of investigating the adultcentric view.

Removing the child from the present and positioning them as a future possibility, from human being to human-becoming, enables the adult, positioned as the ideal, to transfer knowledge and norms, to act upon children so that they can become a “full human being.” As mentioned earlier, this ideal of humans must fulfill the bourgeois expectations and demands, being the adultcentric paradigm, the belief structure that makes possible for Bleak Pedagogical practices to take place when trying to accomplish the “adult destiny.” Possible to relate with the term “Manifest Destiny,” coined by O’Sullivan in 1845 in reference to the US’s to invade and conquer territories and native peoples as a divine order, and Kipling’s White Man’s Burden” (Kipling, 1903), societal common sense has placed adults in the position of child colonizers. The school, as the most widespread formal educational institution, is therefore the setting in which hierarchy and the manifestation of such beliefs can take place. From the emerging relationship between EV literature and Adultcentrism, it follows that Bleak Pedagogy and the adultcentric bias not only make EV possible and justifiable but also predictable.

### 3.3 Future perspectives

Drawing on the above-presented Adultcentrism framework in relation to emotional violence by teachers toward students, this study advances the hypothesis that Adultcentrism is the underlying cause of such violence and is reflected in their (Bleak) pedagogical practices.

The argument developed above moves from concrete, empirical evidence on teacher EV to broader systemic aspects that can explain it. A varied terminology was noted in the literature, while the outcomes for victims seem to have negative implications across studies. Evidence was analyzed on teachers’ and students’ characteristics, but the results were not unanimous. Even when one number was outstanding in results, such as boys’ victimization, the system of beliefs that could explain why this happens was not analyzed.

The dialogue between protection discourse and empirical evidence was analyzed, revealing that current practices fail to protect students, often re-victimizing them and obscuring the bigger picture.

The Bleak Pedagogy concept, regarding the practices and values teachers may carry, was presented as a way to examine this situation without judging its actors, but by looking at a broader aspect. Those pragmatic and paradigmatic beliefs do not reflect individual flaws but rather cultural systems. Within a larger system of beliefs, the adultcentric bias was introduced as a concept that addresses deeper views of who children are and what adult roles must be. At a level that goes beyond conscientious choices, it can explain how EV can be present across countries and cultures.

Following the exploration of theoretical and empirical correlations between EV and BP, and previous studies showing that BP correlates with Adultcentrism bias. This framework suggests that, since much of the perpetrated EV is not recognized by teachers as such, the Adultcentrism bias can be understood as rooted in ongoing cultural norms, without their conscious acknowledgment of the acts and effects of their violent behavior.

Drawing on philosophical bases and perspectives on school violence, such as Freire’s banking education and Bourdieu’s symbolic violence, these concepts can shift from philosophical stances toward a pragmatic explanation of a very concrete situation that children are experiencing. The Adultcentrism perspective can provide a new lens for understanding current findings on victimization by addressing what leads teachers to victimize some student profiles more than others.

The present hypothesis generates the testable predictions that higher scores on teachers’ Bleak Pedagogy (BP) practices and Adultcentric (AD) biases reflect higher emotional victimization reported by students. Also anticipates that teachers fail to identify their BP practices as emotionally violent. It is expected that, as in previous results, a stronger agreement with AD views is correlated with BP practices in different cultural contexts.

These predictions can be tested by a quantitative method, collecting data from students and teachers using established scales, such as the International Society for the Prevention of Child Abuse and Neglect (ISPCAN) Child Abuse Screening Tool Children’s Version (ICAST-C) (Zolotor et al., 2009) for children to report experienced violence, including EV, and the Adultcentrism Scale (Florio et al., 2020a) and Bleak Pedagogy Scale (Florio et al., 2020b) for teachers to answer, and for us to be able to analyze if there is a correlation between the three, and if one is predicted by the other. Intercultural translation and validation of scales can increase comparability of testing and results.

This new understanding of teachers’ EV-underlying bias also points toward possibilities for transforming power relations in schools. As a critical orientation confronting Adultcentrism, childism offers an alternative perspective. A childist educational attitude would ensure space for mutual teaching (Biswas and Mattheis, 2021) while acknowledging the interdependent empowerment of its actors (Wall, 2019). If we accept that public schools carry a childist emancipatory potential (Snir, 2024), challenging the normative power dynamics of schools through childism allows the imagination and development of teacher–student relationships toward greater equity.

## 4 Conclusion

This article aimed to understand the causes and motivations for teacher emotional violence against students. From an inductive logic structure, the definitions, current research, and unanswered questions related to EV in the teacher–student relationship were presented. The theoretical framework of Adultcentrism and Bleak Pedagogy was presented in dialogue with the research gap. While most research to date has focused on children’s characteristics to explore the causes and predictors of such behaviors, this study repositions children and teachers. The idea of an adultcentric bias in adults’ views was proposed as the main cause of violent behaviors. These ideas align with the discussions of power dynamics in emotional violence literature, situating EV from teachers as an implicit societal construction on adult–child relationships, from which emotional violence can be an expected result. To our knowledge, no model to date has connected and tested these perspectives.

The limitations regarding the literature review must be acknowledged. Citation chaining may produce biased results, favoring similar frameworks and highly cited authors, despite attempts to include emerging literature. As with general critical reviews, it lacks systematic results and relies more on subjective interpretation; on the other hand, it serves as a foundation rather than a result (Grant and Booth, 2009). A limitation of the theoretical construction is the reliance on conceptual articulation rather than empirical data, highlighting the need for further research. The hypothesis focuses on teachers’ beliefs and has limitations in fully understanding EV; therefore, future studies may need to consider children’s backgrounds, school climate, and other factors.

Empirical validation of these hypotheses would mean profound implications for policies, practices, and future research. Evidencing the need for a lens shift for adults, implications for teacher training programs, and child protection practices.

Discussing hierarchical school systems, rather than blaming individuals, can open the possibility of mutual emancipation for students and teachers. This conceptual shift regarding the predecessors and predictors of a real, painful problem that children face could open us up to other possibilities in education.

Future research should test the Adultcentrism hypothesis across cultures and nations; historical interactions and over-time alterations should also be evaluated, addressing the chronological systems further proposed by Bronfenbrenner and Morris (1998). Longitudinal studies are also recommended to analyze teacher training programs across different countries, aiming to identify the presence of adultcentric views and their impact on classroom practices. Aspects to consider include the immigrant status of children in European countries, examining ethnocentric control and discipline measures and whether these children experience higher victimization; children’s gender differences and correlations, since girls are more disciplined and controlled in schools, internalizing symptoms, and boys, externalizing and more victimized by their teachers; class differences, to understand if there are differences in teacher–student, school, and nation levels. Listening to families’ and children’s understanding of EV and exploring its nuances and definitions with children are also relevant to understanding EV.

In conclusion, this theoretical contribution reframes the understanding of emotional violence from teachers toward students as a product of societal beliefs. By articulating it alongside Bleak Pedagogy and Adultcentrism, a new framework for empirical testing is presented, enabling new strategies for its recognition and prevention. Addressing these systemic biases is, above all, a call for researchers, educators, and policymakers to move beyond surface-level reflections and seek solutions that fulfill the school’s emancipatory and child-centered potential.

## Data Availability

The original contributions presented in the study are included in the article/supplementary material, further inquiries can be directed to the corresponding author.

## Footnotes

1 The term “teacher bullying” was chosen due to its use in the specific research. Considerations regarding its use should be taken into account.
